# Establishing a Semen Cryobank for Yan Goose: Optimized Collection and Cryopreservation

**DOI:** 10.3390/ani16162457

**Published:** 2026-08-07

**Authors:** Lihua Cao, Lijun Zhu, Bocheng Liu, Weimin Jiang, Hailin Liu, Guitao Jiang, Xiaohua Feng, Chen Chen, Haifeng Yan

**Affiliations:** 1 Hunan Institute of Animal and Veterinary Science, Changsha 410131, China; caolihua@hunaas.cn (L.C.); zhulijun1003@163.com (L.Z.); liubocheng920@hunaas.cn (B.L.); jwm111@hunaas.cn (W.J.); liuhailin@hunaas.cn (H.L.); jiangguitao@hunaas.cn (G.J.); fxh2025@hunaas.cn (X.F.); 2 Yuelushan Laboratory, Changsha 410016, China

**Keywords:** Yan goose, semen cryopreservation, dimethylformamide, dimethylacetamide, teaser-induced semen collection, genetic resource conservation

## Abstract

Yan goose, a representative Chinese local breed, requires genetic resource conservation. To address this, we optimized a semen cryopreservation protocol for Yan geese and validated its feasibility in large-scale cryobank production. Using motility of frozen-thawed sperm (MFS) as the primary indicator, supplemented by artificial insemination (AI) trials, we systematically evaluated cryoprotectant type, freezing rate, extender osmolality and pH, and thawing rate. Dimethylformamide (DMF) significantly outperformed dimethylacetamide (DMA) in MFS (34.17% vs. 24.17%, *p* < 0.05). Based on optimization data and practical requirements, the final DMF method comprised freezing at 2.0 cm above liquid nitrogen for 2 min, extender osmolality of 335 mOsm/kg at pH 7.8, and thawing at 5 °C for 60 s. Teaser-induced semen collection was successfully applied in a Chinese local goose breed for the first time, achieving a 69.14% success rate. Using this system, production over 17 days yielded 1225 straws (96.61% qualified), of which 838 from 30 families were banked. AI of banked semen achieved a fertilization rate of 70.4% and a hatchability of 86.8%. These findings demonstrate the successful establishment of a cost-effective cryobank for Yan geese, providing a feasible paradigm for conserving other Chinese local goose breeds.

## Simple Summary

This study developed a semen cryopreservation protocol for Yan goose, a representative Chinese local breed, and successfully built a large-scale cryobank. The cryopreservation protocol was established using DMF as the cryoprotectant, and the large-scale production was enabled by the application of teaser-induced semen collection. In artificial insemination trials, the banked semen achieved a 70.4% fertilization rate and 86.8% hatchability, demonstrating the functional utility of the cryopreserved genetic material. Over 17 production days, 1225 frozen straws were produced with a 96.61% qualification rate, of which 838 straws from 30 families were banked. This integrated approach can serve as a practical reference for establishing similar conservation programs for other indigenous goose breeds in China.

## 1. Introduction

Due to livestock and poultry breed improvement and the introduction of high-yield exotic breeds, numerous indigenous local breeds have become endangered. Even breeds that are not yet on the verge of extinction face severe conservation challenges once they lose commercial value: in situ conservation incurs sustained high costs and grows increasingly unsustainable long-term. It has been proposed that keeping live populations is cost-effective for periods of up to three years, but becomes difficult to justify beyond three years; for any population that will not be used within five years, cryoconservation should be pursued and in situ maintenance discontinued [1]. Semen cryopreservation enables long-term indefinite storage of sperm in liquid nitrogen [2], rendering it the most practical ex situ conservation strategy for poultry genetic resources [3]. However, geese pose particular challenges owing to seasonal reproduction, complex mating behavior, and high inter-individual variance in semen fertilization rate [4,5,6]. To date, no single freezing protocol suits all poultry species, necessitating breed-specific optimization of cryoprotectants, freezing rates, extenders and thawing procedures [7].

Semen collection in geese remains a major bottleneck for the widespread application of cryopreservation. Manual massage requires dedicated training and elicits a response in only ~50% of ganders [8]; average ejaculate volume is about 0.3 mL, with individual volumes ranging from 0.05 to 0.95 mL [8,9], indicating substantial inter-individual variation. Despite its widespread use, the massage method is labor-intensive, operator-dependent, and often induces stress responses that compromise semen quality. Teaser-induced semen collection can serve as an alternative technique for semen collection in poultry. Teaser goose presence during collection has been shown to improve semen density in waterfowl [10], and housing ganders singly with a teaser goose introduced just before collection improved success rate compared to single housing without a teaser goose or group housing [11]. Similar results have been reported in Sasso cocks [12]. However, the application of teaser-induced semen collection in Chinese local goose breeds has not been systematically reported, and an integrated training and collection regime for large-scale cryobank production remains to be established.

Cryopreservation outcomes are influenced by multiple factors related to breed, individual donor, ejaculate quality, and freezing method [13]. Extender selection is critical, as sperm encounter severe osmotic stress during cryopreservation that impairs motility and fertilizing capacity [14,15]; osmolality extremes cause swelling or shrinkage, while pH affects metabolic rate [15]. The commonly used Lake extender for chicken semen has an osmolality of 340–350 mOsm/kg and pH 7.0–7.2 [15], but optimal values for geese appear breed-dependent: 390 mOsm/kg for White Italian goose [7] and 340 mOsm/kg for Canada goose [16]. Cryoprotectant choice is also critical. Currently, glycerol and dimethylacetamide (DMA) are widely used for poultry semen cryopreservation [17], while dimethylformamide (DMF) has also shown protective effects on gander sperm [13]. However, their efficacy varies across species and protocols, and systematic comparisons between DMA and DMF in goose semen cryopreservation are limited. Freezing rate is typically achieved using programmable freezers or liquid nitrogen vapor fumigation; the latter is simpler and more cost-effective [18], but most existing goose studies have relied on programmable freezers [19]. Our previously developed liquid nitrogen vapor fumigation device has been successfully applied in chicken semen freezing [20], but its applicability to geese remains to be evaluated. For DMA, optimal freezing rates vary widely, ranging from 2 cm × 3 min [21] to 3 cm × 1 min [22] and even 7 cm × 10.5 min [23], reflecting breed- and protocol-specific differences [24,25,26], with some studies employing two-step protocols [26]. For DMF, systematic comparisons are scarcer. In addition, thawing conditions vary across goose breeds, e.g., 60 °C for several seconds for White Italian goose [2] versus 40 °C for 2 min for Landes goose [27,28], indicating that no uniform standard exists for goose semen thawing. Thus, a comprehensive and systematic optimization of these interdependent parameters is essential for developing a reliable cryopreservation protocol for Chinese local goose breeds.

In the field of genetic resource conservation, several recommendations exist regarding the minimum number of donors for cryobanking. Silversides et al. [1] proposed that a minimum of 25 individuals per line should be used as semen donors, and also highlighted the substantial cost advantages of cryopreservation over live conservation. The French poultry gene bank recommends selecting 30 genetically well-characterized individuals as optimal donors for endangered breeds [29]. The FAO suggests that the amount of genetic material collected should meet 200% of the requirement for population restoration (or 150% when no additional research use is anticipated) [30]. Łukaszewicz et al. [31] further emphasized that gene-banked breeds require sufficient offspring to ensure effective population recovery. Despite these guidelines, the establishment of a functional cryobank for Chinese local goose breeds—encompassing not only protocol optimization but also large-scale production, quality control, and systematic repository organization—has rarely been reported. The development of a simple, efficient and low-cost cryobank system for geese remains an urgent priority.

Yan goose, originated in the Lu’an area of Anhui Province, is one of the rare medium-sized grey goose breeds in China and a classic example of the conservation dilemma described above. It was twice listed in the National List of Protected Livestock and Poultry Genetic Resources (in 2000 and 2006), but was removed from the national list in 2014 due to its small population size, limited market demand for grey feathers, and low reproductive rate. It is currently maintained only as a provincial-level protected breed in a single conservation farm in Anhui Province through live conservation. This status underscores the urgent need for a cost-effective semen cryobank to safeguard its genetic resources for future utilization. In this study, we used Yan goose as the experimental model to systematically optimize cryopreservation parameters (cryoprotectant selection, freezing rate, extender osmolality and pH, and thawing procedure) and to apply the optimized protocol to establish a large-scale cryobank encompassing teaser-induced semen collection, multi-tier quality control, AI validation, and final repository organization with 30 families. This study aims to provide a comprehensive and practical cryobanking framework that can serve as a reference for the conservation of other Chinese local goose breeds facing similar challenges.

## 2. Materials and Methods

### 2.1. Birds

All Yan ganders were sourced from the floor-rearing population of the National Waterbird Gene Bank. They were used in two separate batches: 30 ganders were introduced at 8 months of age for protocol optimization; the other 66 ganders were introduced as one-month-old goslings, reared to sexual maturity (8 months of age), and used for large-scale cryobank production. For AI trials, 30 healthy Sichuan White goose females at 14 months of age were provided by the Wugang Tong goose resource farm in Hunan Province. Birds were reared in individual cages under natural light and fed ad libitum with a commercial diet procured from Changsha Huagang Feed Co., Ltd. (Changsha, Hunan, China). The diet contained crude protein 16.54%, metabolizable energy 11.43 MJ/kg, crude fiber 5.35%, calcium 0.71%, and available phosphorus 0.42%. All animal procedures were conducted in strict accordance with the guidelines approved by the Animal Care and Use Committee of the Hunan Institute of Animal and Veterinary Medicine (Approval No. 20231024).

### 2.2. Semen Collection

Semen was collected using two approaches depending on the purpose. The massage method was used during the early-stage optimization experiments, whereas teaser-induced semen collection was adopted for large-scale cryobank production to improve collection efficiency. For optimization, semen was collected from 30 ganders via dorso-abdominal massage [32] after 9 days of cage adaptation and 14 days of training. For cryobank production, 66 ganders underwent 7 days of direct teaser training (without prior massage); a teaser goose was introduced and sexual responses (head-pecking, tail raising, and mounting) were observed within 2 min as indicators of training efficacy. Ganders that ejaculated cleanly on three consecutive attempts were qualified as donors. For both methods, only ejaculates free of fecal or urinary contamination were retained. Ejaculate volume was recorded, and semen was immediately diluted with pre-warmed extender at room temperature. The dilution ratio was 1 part semen to 0.5–1 part extender for massage-collected semen, and 1:2 for teaser-induced semen. Diluted semen was transported to the laboratory within 30 min in a portable cooler at 5 °C.

### 2.3. Assessment of Sperm Motility

Sperm motility was assessed using a video thermostatic microscope (BH-2, Shangrao Tiance Optical Instrument Co., Ltd., Shangrao, Jiangxi, China) at a total magnification of 200× and a constant temperature of 32 °C. A 10 µL aliquot of diluted or thawed semen was placed on a pre-warmed slide, and the average motility from five random fields was recorded. Motility was defined as the percentage of progressively motile spermatozoa across five randomly selected microscopic fields per sample. Each sample was assessed in triplicate by the same trained observer, who was blinded to the treatment groups. Sperm motility before freezing (SMF) and MFS were recorded accordingly.

### 2.4. Freezing Procedure

Diluted semen was equilibrated at 4 °C for 15 min, and then DMA or DMF (Sigma-Aldrich, St. Louis, MO, USA) was added to a final concentration of 6% (*v*/*v*), a concentration previously utilized for gander semen cryopreservation [2,7]. The semen was aspirated into 0.25 mL straws (IMV, L’Aigle, France) and sealed with polyvinylpyrrolidone (PVP) powder (Sigma-Aldrich). Liquid nitrogen was poured into the container 10 min prior to use to a depth of 5 cm. Straws were frozen by exposure to liquid nitrogen vapor at the specified height and duration, then plunged into liquid nitrogen (−196 °C). Freezing rate was expressed as fumigation height × duration (cm × min). MFS was used as the primary evaluation indicator. Unless otherwise specified, all screenings were conducted with thawing at 5 °C for 60 s.

### 2.5. Determination of Optimal Freezing Rate

For the DMF-based method, the optimal freezing rate was screened in two stages. In the first stage, straws were frozen at 0.5, 1.5 and 2.0 cm above the liquid nitrogen surface for 2 min. In the second stage, straws were frozen at 2.0, 3.0 and 4.0 cm for 2 min. For the DMA-based method, straws were frozen at 0.5, 1.5 and 2.0 cm above the liquid nitrogen surface for 2 min. Each screening experiment was performed with 4 replicates using pooled semen from 5 ganders.

### 2.6. Screening of Extender Osmolality and pH

Semen extenders were formulated based on published literature [2,20] with minor modifications; compositions are presented in Table 1. For each cryoprotectant, the optimal extender formulation was screened using the optimal freezing rate determined in Section 2.5. For the DMF-based method, osmolality was screened using extenders E and E2, followed by pH screening using E2, E6 and E8. For the DMA-based method, osmolality was screened using extenders R and R1 followed by pH screening using R, R2, R3 and R4. MFS was used as the evaluation indicator. The number of replicates and the pooling scheme were the same as described in Section 2.5.

### 2.7. Comparison of DMA and DMF Freezing Methods Using Their Respective Optimized Protocols

Using the respective optimal parameters for each cryoprotectant, the DMA and DMF freezing methods were compared using split aliquots from the same pooled Yan goose semen sample. MFS was used as the evaluation indicator. The number of replicates and the pooling scheme were the same as described in Section 2.5.

### 2.8. Screening of Thawing Procedure

Thawing rate, expressed as water bath temperature × thawing duration (°C × s), was optimized by testing three rates: 5 °C × 60 s, 40 °C × 10 s, and 60 °C × 5 s, using MFS as the primary indicator. To further assess the effect of thawing rate on fertility, DMF-frozen semen was thawed at 5 °C × 60 s or 60 °C × 5 s and inseminated (Section 2.11), with fertilization rate and hatchability as the fertility indicators. The number of replicates and the pooling scheme were the same as described in Section 2.5.

### 2.9. Large-Scale Cryobank Production

For large-scale production, semen from each individual gander was processed separately without pooling, using the teaser-induced semen collection method and the optimized DMF freezing protocol. Production was conducted over 17 consecutive working days. On each production day, semen was collected from qualified donors, diluted, mixed with cryoprotectant, packaged into 0.25 mL straws, and frozen. Ten minutes after freezing, randomly selected straws were thawed to assess MFS; straws with motility ≥ 20% were considered qualified. Qualified straws from the same gander (family) were pooled into one cryovial (maximum 25 straws per vial) and stored in fabric bags submerged in liquid nitrogen. Each cryovial and bag was labeled with breed, family number, production date, and straw count, with detailed documentation maintained for each family.

### 2.10. Quality Control of Cryopreserved Semen

A multi-tier quality control system was established for the cryobank production: (1) gross inspection at collection for color, consistency and contamination; (2) pre-freeze quality assessment: after dilution, SMF was assessed, and sperm density was routinely estimated by comparison with standard reference images (visual density); (3) MFS assessment of randomly selected straws from each batch, thawed at 5 °C for 60 s; and (4) random sampling for bacteriology and precise counting: For each batch, 3 straws were randomly selected. Serial dilutions (10^−1^, 10^−2^, 10^−3^) were prepared in sterile water and plated on nutrient agar; colonies were counted after 48 h at 37 °C. Sterile water and blank medium served as negative controls. For quality control validation, sperm density was determined using a haemocytometer after dilution with 3% NaCl at a 100 × dilution (counted density): density (×10^8^/mL) = (sperm count in 5 squares) × 5 × 10^4^ × dilution factor. Functional validation of semen quality was performed via AI trials.

### 2.11. Artificial Insemination

AI was conducted to validate the fertility of cryopreserved semen using 30 healthy Sichuan White goose females at 14 months of age. The females were divided into three groups of 10 birds each. All females were at a comparable stage of lay at the onset of the trial. All Inseminations were performed at the same time of day by the same inseminator. For the thawing temperature comparison, frozen straws were prepared from the same batch of pooled fresh semen and aliquoted before freezing, ensuring identical semen source and sperm dose between the two treatment groups. Two separate AI trials were performed: one compared thawing temperatures (5 °C for 60 s vs. 60 °C for 5 s) using two of the groups; the other validated banked semen fertility using the third group, for which straws from different families were randomly selected, thawed at 5 °C for 60 s, pooled, and reassessed for MFS and density before insemination. Semen was inseminated directly after thawing, without any dilution or removal of the cryoprotectant. In both trials, each female received 0.17 mL of thawed semen per insemination, containing approximately 1 × 10^8^ spermatozoa, deposited approximately 5 cm into the oviduct using a 1 mL syringe. In the thawing temperature trial, females were inseminated twice; in the banked semen validation trial, females were inseminated three times. The cloaca was everted and the oviduct opening located; syringes were wiped with saline-soaked cotton after each use and replaced between groups. Eggs were collected daily from 24 h after the first insemination for 13 days; eggs laid within the first 24 h were discarded. Each egg was labeled, fumigated, and incubated. Fertility was determined by candling on day 15, and hatchability was recorded after hatching. Fertilization rate was calculated as the number of fertile eggs divided by the total number of eggs incubated; hatchability was calculated as the number of goslings hatched divided by the number of fertile eggs.

### 2.12. Statistical Analysis

All data are presented as means ± *SEM*. Normality and homogeneity of variance were assessed using Shapiro–Wilk and Bartlett tests, respectively, before applying parametric analyses. Statistical analyses were performed using R software (version 4.3.0, R Core Team, Vienna, Austria, 2024). Comparisons between two groups were analyzed using Student’s *t*-test, and comparisons among multiple groups by one-way ANOVA followed by Tukey’s HSD post hoc test where appropriate. For *t*-tests, exact degrees of freedom and *p*-values are provided in table notes. For ANOVA, overall degrees of freedom (*df*) and *p*-values are reported in table notes, and significant differences (*p* < 0.05) in pairwise comparisons are indicated by different superscript letters within each row or column.

## 3. Results

### 3.1. Characteristics of Fresh Semen and Ganders’ Reaction to Massage Training

After 14 days of daily abdominal massage training, 17 out of 30 ganders (56.7%) displayed satisfactory sexual reflexes. The average ejaculate volume was 0.23 mL (range: 0.1–0.7 mL; Supplementary Tables S1 and S2).

### 3.2. Screening of Freezing Rate

As shown in Table 2, the optimal freezing rate differed between cryoprotectants. For the DMF method, MFS at 2.0 cm × 2 min was significantly higher than at 0.5 and 1.5 cm (*p* < 0.05), but similar to 3.0 and 4.0 cm (*p* > 0.05). For the DMA method, MFS at 0.5 cm × 2 min significantly exceeded that at 1.5 cm and 2.0 cm (*p* < 0.05).

### 3.3. Screening of Extender Conditions

As shown in Table 3, for the DMF method, extender osmolality significantly affected MFS, with 335 mOsm/kg (E2) yielding higher values than 415 mOsm/kg (E) (*p* < 0.05). Subsequent pH screening at 335 mOsm/kg showed that MFS at pH 7.8 (E8) was significantly higher than at pH 6.8 (E6) (*p* < 0.05), but not significantly different from pH 7.3 (E2). The E8 extender (pH 7.8) was selected for large-scale production, as it yielded the highest MFS numerically and was therefore adopted for cryobank establishment. For the DMA method, osmolality had no significant effect on SMF or MFS (*p* > 0.05), though 315 mOsm/kg (R) was numerically superior. Subsequent pH screening revealed no significant differences in MFS among pH 6.7, 7.1, 7.4 and 7.8 (*p* > 0.05), with pH 7.1 (R) showing mildly higher values. The optimal DMA extender was therefore 315 mOsm/kg at pH 7.1 (R).

### 3.4. Comparison of Freezing Methods

As shown in Table 4, MFS was significantly higher for the DMF method than for the DMA method (*p* < 0.05).

### 3.5. Screening of Thawing Rate

For both cryoprotectant systems, thawing at 60 °C × 5 s yielded significantly higher MFS than 5 °C × 60 s and 40 °C × 10 s (*p* < 0.05). However, AI trials with DMF-frozen semen showed that the 5 °C × 60 s protocol achieved higher fertilization rate and hatchability than 60 °C × 5 s, despite its lower MFS (Table 5).

### 3.6. Efficacy of Teaser-Induced Semen Collection in Ganders

**Figure 1:**
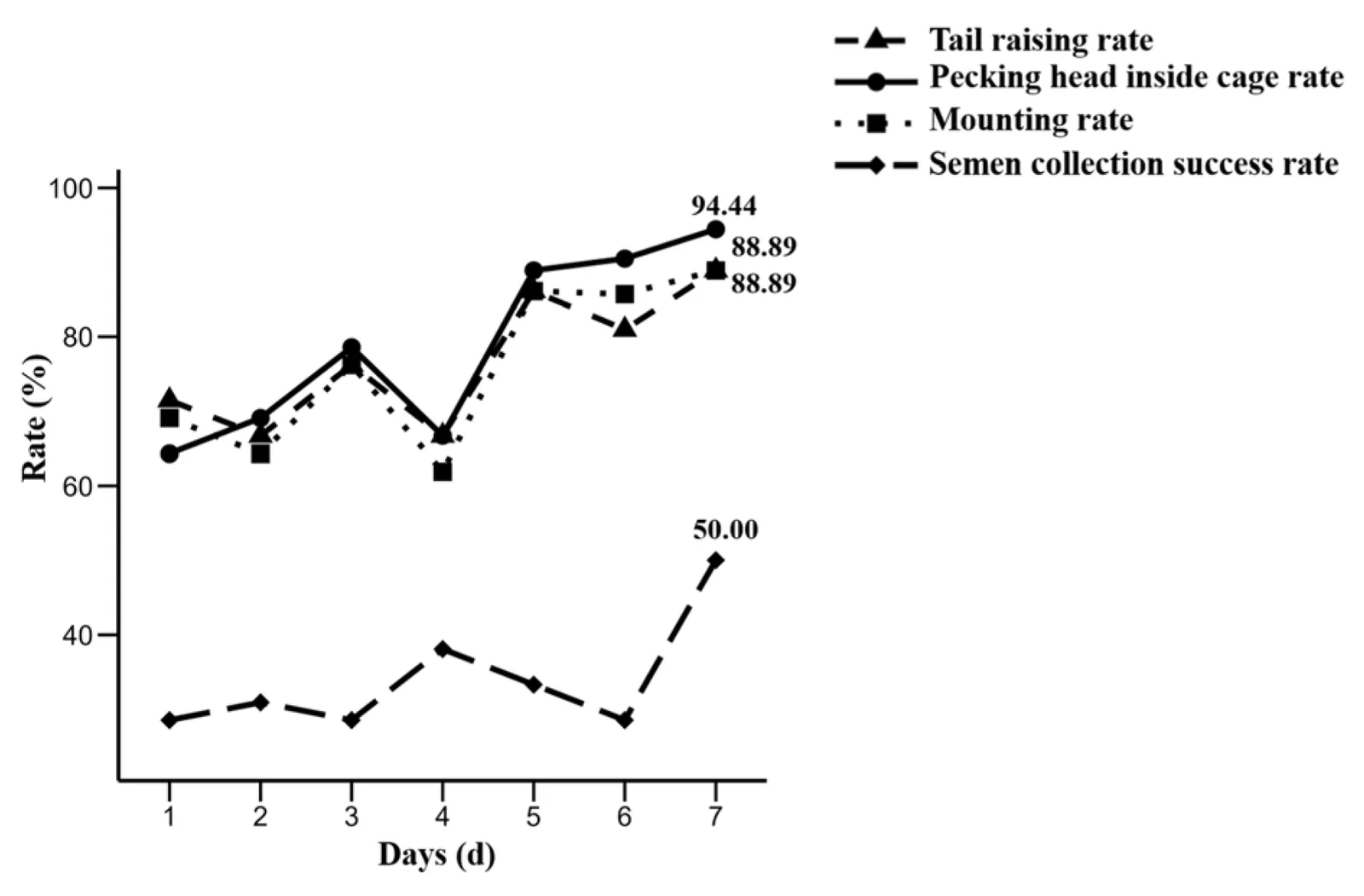
Temporal changes in the efficacy of teaser-induced training in ganders (day 7 data shown).

As shown in Figure 1, after 7 days of teaser training, the rate of head-pecking inside the cage in ganders reached 94.44%, while both tail raising and mounting rates attained 88.89%. The semen collection success rate was 50%.

### 3.7. Overall Quality of Frozen Semen During Large-Scale Cryobank Production

As summarized in Table 6, the mean raw ejaculate volume was 0.25 mL, and the mean SMF and MFS were 20.76% and 24.22%, respectively. The overall qualified straw rate reached 96.61%, with daily rates ranging from 86.21% to 100%.

### 3.8. Cryobank Production Performance and Laboratory Quality Control

A total of 66 ganders were subjected to teaser-induced training, with 186 out of 269 collection attempts successful (69.14%). Fifty-two out of 66 ganders (78.79%) were identified as qualified semen donors. The overall qualified straw rate was 96.61%, with a maximum of 97 straws banked on a single day. On average, 0.15 collection attempts were required per qualified straw. The average daily output per operator was 37.29 total straws and 36.03 qualified straws. The entire production process was completed within 17 consecutive days. Laboratory inspection of 12 randomly selected straws revealed a post-thaw sperm density of 5.96 ± 1.21 × 10^8^ sperm/mL and a total bacterial count of 4.29 ± 1.27 × 10^4^ CFU/mL. AI trials using randomly selected straws from the cryobank yielded a fertilization rate of 70.4% and a hatchability of 86.8% (Table 7).

### 3.9. Overall Semen Quality and Storage Inventory Across Conservation Families

A total of 838 qualified straws were banked from the 30 families, with an average of 28 straws per family. The mean raw ejaculate volume was 0.25 mL, and the mean SMF and MFS were 20.76% and 24.22%, respectively, with corresponding density of 9.93 and 5.69 × 10^8^/mL (Table 8).

## 4. Discussion

In this study, we systematically screened and optimized a semen cryopreservation method for Yan geese—covering cryoprotectant selection, freezing rate, extender osmolality and pH, and thawing procedure—and further validated its applicability in large-scale cryobank production.

### 4.1. Optimization of Cryopreservation Parameters for Yan Goose Semen

Manual massage is the most commonly used method for semen collection in geese. In this study, 56.7% (17/30) of Yan ganders responded after 14 days of training, with an average ejaculate volume of 0.23 mL (range: 0.1–0.7 mL), consistent with the ~50% response rate and confirming substantial inter-individual variation as previously reported [8,9].

Freezing rate critically affects post-thaw sperm quality. The optimal rate for the DMF method (2 cm × 2 min) was consistent with that reported for capercaillie semen [21], whereas the optimal rate for the DMA method (0.5 cm × 2 min) differed from previously reported DMA rates [22,23]. This divergence likely reflects differences in membrane permeability and toxicity between the two cryoprotectants [13,32]: DMF, which has higher membrane permeability and lower toxicity, may benefit from a slower cooling rate to minimize intracellular ice formation, whereas DMA may require more rapid cooling for better post-thaw survival. These results also support the consensus that tailored freezing protocols are necessary for different poultry breeds and cryoprotectants [33]. When the two optimized protocols were compared, the DMF method significantly outperformed the DMA method, consistent with the growing preference for DMF over DMA in avian cryopreservation [31].

Extender osmolality and pH also played critical roles. In this study, for the DMF method, 335 mOsm/kg significantly outperformed 415 mOsm/kg, and pH 7.8 was superior to pH 6.8. For the DMA method, 315 mOsm/kg at pH 7.1 was optimal, though no significant differences were observed among the tested gradients, indicating greater robustness of the DMA system across a wider range of conditions. The optimal osmolality values (315–335 mOsm/kg) are similar to the 340 mOsm/kg reported for Canada goose [16], but substantially lower than the 390 mOsm/kg for White Italian goose [7], suggesting that Chinese local goose breeds may favor relatively lower osmolality. The optimal pH of 7.8 for the DMF method is consistent with the EK extender reported by Łukaszewicz [13]. The pH 7.8 formulation (E8) was therefore selected for large-scale cryobank production, as it consistently yielded the highest MFS.

The thawing procedure proved to be the most nuanced parameter. Notably, the relationship between MFS and fertility is method-dependent: across different thawing methods, higher motility does not always predict higher fertility. In our in vitro assays, 60 °C × 5 s yielded the highest MFS, consistent with reports for White Italian goose [2]. However, AI trials revealed a critical dissociation: the 5 °C × 60 s protocol, despite lower MFS, achieved higher fertilization rate and hatchability (73.81% and 74.19%, respectively) than 60 °C × 5 s (57.14% and 55.00%, respectively). Studies on rooster semen have also shown that thawing at 5 °C can yield superior post-thaw sperm quality compared to higher temperatures [34,35]. A plausible explanation is that rapid thawing may preserve motility but cause sublethal acrosomal or mitochondrial damage, whereas slower thawing at 5 °C may permit gradual repair of osmotic injury, retaining more functionally competent spermatozoa [33]. The 5 °C × 60 s protocol was selected because 60 s is the minimum duration at this temperature that ensures complete thawing of the 0.25 mL straw. Notably, 5 °C is the short-term storage temperature for poultry semen and allows for thawing without specialized equipment (ice packs suffice), offering operational advantages for routine cryobank application. Accordingly, we adopted the 5 °C × 60 s protocol, prioritizing functional fertility over MFS metrics—a principle that should guide protocol optimization when laboratory and fertility assessments diverge. It should be acknowledged that additional parameters such as membrane and acrosome integrity were not systematically evaluated. However, sperm motility is the most practical and widely used parameter for routine quality assessment in poultry semen cryopreservation, and it shows a strong correlation with fertilizing capacity in most contexts. Moreover, the successful AI outcomes confirmed the biological relevance of our motility-based optimization.

### 4.2. Large-Scale Cryobank Production and Practical Considerations for Yan Geese

In the present large-scale cryobank production, we adopted teaser-induced semen collection without prior massage training. After seven days of training, the successful collection rate reached 69.14% (186/269), and 78.79% (52/66) of the trained ganders qualified as regular donors—higher than the 56.7% (17/30) achieved with the massage-only method, likely reflecting the more natural mating stimulus provided by the teaser goose [11]. Notably, semen volume and quality are influenced by environmental temperature, collection frequency and technician skill [8]; therefore, trained and relatively fixed personnel are advisable for routine collection.

The average raw ejaculate volume (0.25 mL) was similar to that obtained by massage, and although lower than the highest international report (0.41 mL, [36]), the average pre-freeze sperm density (9.93 × 10^8^ sperm/mL; ~29.79 × 10^8^ sperm/mL in raw semen) was approximately 3.2-fold higher than the highest reported value (9.27 × 10^8^ sperm/mL, [9]), substantially improving the efficiency of genetic unit banking, an effect likely attributable to the teaser-induced method [10]. Combined with the optimized DMF method, the production trial yielded 1225 qualified straws with an overall qualified rate of 96.61% over 17 working days, confirming the scalability of this integrated system.

For quality control, we established a multi-tier system comprising gross inspection, pre-freeze assessment, post-thaw assessment, and random sampling for bacteriology and AI validation. Sperm motility is universally adopted for evaluating poultry semen quality [37], as it can be detected simply and conveniently in practice [13]. In the AI trial, DMF-frozen semen achieved a fertilization rate of 70.4% (38/54) and a hatchability of 86.8% (33/38). These values are close to the 83.78% fertilization rate and 88.17% hatchability reported for White Italian goose [13], and the fertilization rate is superior to the 58.5% reported by Váradi et al. [27]. The bacterial load averaged 4.29 × 10^4^ CFU/mL, reflecting unavoidable cloacal contamination in poultry and highlighting the need for poultry-specific bacteriostatic techniques. No unified standard exists for bacterial counts in poultry semen. Despite this level, the fertility validation trial achieved a 70.4% fertilization rate, confirming that the observed bacterial load did not impair fertilizing capacity under our experimental conditions. In terms of production efficiency, this integrated system required only 0.15 collection attempts per qualified straw, and each operator produced 36.03 qualified straws daily.

It should be acknowledged that the use of pooled semen during protocol optimization may mask inter-individual variation in sperm freezability. However, during the subsequent large-scale production, all straws were produced from individual ganders, and ganders with acceptable pre-freeze quality consistently yielded acceptable post-thaw quality, confirming the general applicability of the optimized protocol to individual donors. For final repository organization, we banked 838 qualified straws from 30 families, averaging 28 straws per family (range 18–48). This meets international recommendations: 25–30 donors per breed and 150–200% of the minimum required straws for population restoration [1,29,30,31]. Genetic characterization of individual donors was not performed in this study, as the primary goal of this cryobank is to serve as a long-term strategic reserve for the breed. The selection of 30 donors was based on these same recommendations to capture maximal genetic diversity, and each donor’s semen was processed and stored separately to preserve individual genetic contributions. Based on these principles, we propose that approximately 800–1000 straws per breed constitute a cost-effective conservation unit. All straws were stored in 46 cryovials and 15 bags with pedigree records and freezing documentation, ensuring traceability. Taken together, this study demonstrates that a simple, efficient and low-cost cryobank can be successfully established for Yan geese, and the protocols developed here provide a feasible paradigm for the establishment of cryobanks for other Chinese local goose breeds.

## 5. Conclusions

This study demonstrates that a cost-effective cryobank can be established for a representative Chinese local goose breed through systematic protocol optimization and the innovative application of teaser-induced semen collection. The successful AI validation confirms the practical utility of the banked semen. This integrated framework may serve as a useful reference for conserving genetic resources of other indigenous waterfowl breeds facing similar challenges.

## Figures and Tables

**Table 1:** The composition of extenders used for goose semen.

Composition	E	E2	E6	E8	R	R1	R2	R3	R4
Bi-distilled water (mL)	100.00	100.00	100.00	100.00	100.00	100.00	100.00	100.00	100.00
D-fructose (g)	0.20	0.20	0.20	0.20	—	—	—	—	—
Glucose (g)	0.70	0.70	0.70	0.70	—	—	—	—	—
D-glucose-monohydrate (g)	—	—	—	—	0.80	0.80	0.80	0.80	0.80
Potassium citrate (g)	0.14	0.14	0.14	0.14	—	—	—	—	—
Potassium acetate (g)	—	—	—	—	0.50	0.50	0.50	0.50	0.50
Sodium dihydrogen phosphate (g)	0.21	0.21	0.21	0.21	—	—	—	—	—
Di-sodium hydrogen phosphate (g)	0.98	0.98	0.98	0.98	—	—	—	—	—
Magnesium acetate tetrahydrate (g)	—	—	—	—	0.08	0.08	0.08	0.08	0.08
L-Sodium glutamate (g)	1.40	1.40	1.40	1.40	1.92	1.92	1.92	1.92	1.92
Inositol (g)	0.70	0.70	0.70	0.70	—	—	—	—	—
PVP (g)	0.10	0.10	0.10	0.10	0.30	0.30	0.30	0.30	0.30
protamine sulfate (g)	0.02	0.02	0.02	0.02	—	—	—	—	—
Osmolality (mOsm/kg)	415	335	335	335	315	280	315	315	315
pH	7.3	7.3	6.8	7.8	7.1	7.1	6.7	7.4	7.8

Note: All extenders were supplemented with penicillin and streptomycin at 1000 IU/mL each. A dash “—” denotes the absence of the stated component in the extender.

**Table 2:** Screening of freezing rate for DMF and DMA freezing methods, respectively.

Cryoprotectants	SMF (%)	MFS (%) at Different LN_2_ Vapor Height (cm)					
		0.5	1.0	1.5	2.0	3.0	4.0
DMF	33.33 ± 2.89	11.67 ± 2.58 ^c^	—	16.67 ± 4.08 ^b^	26.67 ± 2.58 ^a^	—	—
	37.50 ± 10.61	—	—	—	30.00 ± 5.77	28.75 ± 7.50	20.00 ± 5.77
DMA	36.67 ± 2.89	24.17 ± 3.76 ^a^	14.17 ± 3.76 ^b^	—	11.67 ± 2.58 ^b^	—	—

Note: All freezing trials were conducted with a 2 min exposure to liquid nitrogen vapor. DMF screening was conducted in two independent experiments: Exp 1 compared 0.5, 1.5 and 2.0 cm (*df* = 2, 9; *p* < 0.001); Exp 2 compared 2.0, 3.0 and 4.0 cm (*df* = 2, 9; *p* = 0.451). DMA screening compared 0.5, 1.0 and 2.0 cm (*df* = 2, 9; *p* = 0.021). Different superscript letters within the same row indicate significant differences among vapor heights (*p* < 0.05). Dashes (—) indicate conditions not tested.

**Table 3:** Screening conditions of semen extenders.

Cryoprotectants	Extender	Osmolality (mOsm)	pH	SMF (%)	MFS (%)
DMF	E	415	7.3	31.67 ± 2.89	21.11 ± 2.21 ^b^
	E2	335	7.3	35.00 ± 0.00	30.00 ± 2.50 ^a^
DMA	R	315	7.1	27.83 ± 5.67	21.17 ± 3.76
	R1	280	7.1	22.33 ± 4.46	18.00 ± 5.66
DMF	E2	335	7.3	34.17 ± 3.76	20.00 ± 0.00 ^ab^
	E6	335	6.8	33.33 ± 5.16	17.50 ± 2.89 ^b^
	E8	335	7.8	30.83 ± 5.85	22.50 ± 2.89 ^a^
DMA	R	315	7.1	25.71 ± 6.73	16.71 ± 6.63
	R2	315	6.7	24.00 ± 2.24	16.00 ± 4.18
	R3	315	7.4	23.57 ± 4.76	15.29 ± 4.72
	R4	315	7.8	20.71 ± 6.08	14.29 ± 3.45

Note: Osmolality effects were assessed by paired comparisons (E vs. E2 for DMF; R vs. R1 for DMA). pH effects were assessed within each osmotic condition (E2/E6/E8 for DMF; R/R2/R3/R4 for DMA). Different superscript letters within each comparison group indicate significant differences (*p* < 0.05), absence of letters indicates no significant difference (*p* > 0.05). Same below. Statistical details: DMF osmolality (E vs. E2, *t*-test: *df* = 6; *p* = 0.023); DMF pH overall (ANOVA: *df* = 2, 9; *p* = 0.064); DMF pH 7.8 vs. 6.8 (Tukey’s HSD: *df* = 9; *p* = 0.044); DMA osmolality (R vs. R1, *t*-test: *df* = 6; *p* = 0.375); DMA pH overall (ANOVA: *df* = 3, 12; *p* = 0.838).

**Table 4:** Comparison of MFS in two freezing methods.

Freezing Method	SMF (%)	MFS (%)
DMA	36.67 ± 2.89	24.17 ± 3.76 ^b^
DMF	43.33 ± 5.77	34.17 ± 2.04 ^a^

Different superscript letters within the same row indicate significant differences among vapor heights (*p* < 0.05).

**Table 5:** Screening of thawing rate for frozen semen.

Temperature (°C)	Time (s)	MFS of DMA Semen (%)	MFS of DMF Semen (%)	Fertilization Rate of DMF Semen (%)	Hatchability of DMF Semen (%)
5	60	19.75 ± 3.43 ^b^	21.11 ± 2.66 ^b^	73.81 (31/42)	74.19 (23/31)
40	10	21.50 ± 3.29 ^b^	21.11 ± 2.20 ^b^	—	—
60	5	24.75 ± 5.14 ^a^	31.11 ± 2.55 ^a^	57.14 (20/35)	55.00 (11/20)

Note: MFS comparisons among thawing rates: DMA (*df* = 2, 9; *p* = 0.023); DMF (*df* = 2, 9; *p* < 0.001). Dashes (—) indicate conditions not tested. Different superscript letters within the same row indicate significant differences among vapor heights (*p* < 0.05).

**Table 6:** Overall quality of frozen semen produced for the Yan goose cryobank over consecutive production days.

Index	Mean ± *SEM*	Range
Raw ejaculate volume (mL)	0.25 ± 0.15	0.13–0.34
SMF (%)	20.76 ± 4.99	15.27–23.89
Pre-freeze sperm density (×10^8^/mL)	9.93 ± 2.31	8.86–11.55
MFS (%)	24.22 ± 4.31	21.42–27.14
Post-thaw sperm density (×10^8^/mL)	5.69 ± 1.42	5.00–6.55
Number of qualified straws per day	72.06 ± 27.92	25–114
Overall qualified straw rate (%)	96.61 (1225/1268)	86.21–100

Note: Data are derived from daily records over 17 consecutive production days (*n* = 17). Full daily records are available in Supplementary Table S3. Pre-freeze density was estimated by visual assessment; post-thaw density was determined by haemocytometer counting.

**Table 7:** Production efficiency and microbial inspection results of cryopreserved semen from Yan geese for germplasm conservation.

Category	Indicators	Value
Production efficiency	Number of trained ganders	66
	Successful semen collection rate (%)	69.14 (186/269)
	Ratio of qualified ganders (%)	78.79 (52/66)
	Overall qualified straw rate (%)	96.61 (1225/1268)
	Maximum daily banked straws	97
	Average collection times per qualified straw	0.15 (186/1225)
	Average total straws per person per day	37.29
	Average qualified straws per person per day	36.03
	Total production duration (d)	17
Laboratory inspection	Post-thaw sperm density (×10^8^/mL)	5.96 ± 1.21
	Total bacterial count (×10^4^ CFU/mL)	4.29 ± 1.27
Fertility trial	Fertilization rate (%)	70.4% (38/54)
	Hatchability (%)	86.8% (33/38)

Note: Twelve frozen straws were randomly selected from different families for density and bacterial assays. “Maximum daily banked straws” refers to the highest number of straws actually preserved on a single day; this value may differ from total qualified straws produced that day, as some were used for quality testing.

**Table 8:** Overall semen quality and storage inventory of cryopreserved semen from 30 Yan goose conservation families.

Items	Mean ± *SEM*	Range
Raw ejaculate volume (mL)	0.25 ± 0.15	0.12–0.63
SMF after dilution (%)	20.76 ± 4.99	14.50–25.00
Pre-freeze sperm density (×10^8^/mL)	9.93 ± 2.31	8.00–14.00
MFS of qualified frozen semen (%)	24.22 ± 4.31	20.00–30.50
Post-thaw sperm density (×10^8^/mL)	5.69 ± 1.42	3.50–8.00
Average frozen straws per family	28	18–48
Total qualified frozen straws banked in cryobank	838	—
Total cryovials for storage	46	—

Note: Individual data for each of the 30 families are provided in Supplementary Table S4. All semen was cryopreserved using the optimized DMF method. Of the 1225 qualified straws produced, 838 were retained for the final 30-family cryobank; the remainder were used for quality control and AI trials.

## Data Availability

Data is contained within the article or Supplementary Materials.

## Supplementary Materials

The following supporting information can be downloaded at: https://www.mdpi.com/article/10.3390/ani16162457/s1, Table S1: Training progress of massage-based semen collection in Yan ganders; Table S2: Characteristics of massage-based semen collection; Table S3: Daily production quality of frozen semen during Yan goose cryobank establishment; Table S4: Family-level semen quality and storage inventory for the Yan goose cryobank (30 families).

## Abbreviations

The following abbreviations are used in this manuscript:

SMF	Sperm motility before freezing
MFS	Motility of frozen-thawed sperm
AI	Artificial insemination
