# Supplementary material for: Establishing a Semen Cryobank for Yan Goose: Optimized Collection and Cryopreservation
Source: Animals (Basel). 2026 Aug 7;16(16):2457. doi: 10.3390/ani16162457 (PMC13508898; doi:10.3390/ani16162457)
Supplement: Supplementary file 1 [file animals-16-02457-s001.zip › animals-4453273-supplementary.pdf]

Supplementary tables

Table S1. Training progress of massage-based semen collection in Yan ganders.

Training days (d)	Tail raising (%)	Grade A (%)	Grade B (%)	Grade C (%)	Natural penis eversion (%)	Favorable sexual reflex (%)
1	96.67	23.33	10	20	23.33	—
2	66.67	40	10	6.67	10	—
3	66.67	50	6.67	6.67	10	—
4	56.67	33.33	10	13.33	6.67	—
5	60	43.33	10	6.67	13.33	—
6	60	36.67	3.33	20	3.33	—
7	50	30	3.33	16.67	0	—
8	46.67	30	10	6.67	6.67	—
9	50	30	13.33	6.67	10	—
10	63.33	43.33	16.67	3.33	16.67	—
11	63.33	30	6.67	6.67	23.33	—
12	46.67	40	3.33	3.33	26.67	—
13	60	23.33	3.33	6.67	13.33	—
14	73.33	40	23.33	10	13.33	—
Total	—	—	—	—	—	56.67

Notes: A total of 30 Yan ganders were consistently used throughout the 14-day training period. All percentage values were calculated as (number of ganders with positive response / 30) × 100 and rounded to two decimal places. Grades A, B and C represent distinct degrees of cloacal tumescence evaluated during massage: Grade A, obvious and rigid cloacal tumescence with short massage duration; Grade B, prominent yet moderately firm cloacal tumescence after prolonged massage; Grade C, indistinct and flaccid cloacal tumescence even with extended massage. The "Favorable sexual reflex" column represents the cumulative percentage of ganders that exhibited Grade A response or natural penis eversion by the end of the training period (56.67%, i.e., 17/30). Dashes (—) indicate not applicable.

Table S2. Characteristics of massage-based semen collection.

Semen collection round	No. of males (n)	No. of ejaculated males (n)	Ejaculate volume (mL)
The first round		7	0.20±0.10
second round	30	9	0.26±0.12
third round		7	0.26±0.21
total	—	—	0.23±0.14

Notes: Semen was collected via dorso-abdominal massage after 14 days of training. Ejaculate volume is presented as mean ± SEM. Dashes (—) indicate not applicable.

Table S3. Daily production quality of frozen semen during Yan goose cryobank establishment.

Production day	Raw ejaculate volume (mL)	SMF (%)	Pre-freeze concentration (×10⁸ /mL)	MFS (%)	Post-thaw concentration (×10⁸ /mL)	Qualified straw rate (%)
1	0.15±0.06	23.89±5.56 abc	9.56±1.88	25.00±4.33 ab	5.44±1.42	100% (41/41)
2	0.14±0.03	23.14±4.91 abc	11.00±2.71	27.14±3.18 ab	6.00±0.58	87.50% (28/32)
3	0.17±0.05	23.25±3.11 abc	10.50±1.38	26.83±7.03 ab	6.17±1.11	96.49% (55/57)
4	0.13±0.04	21.25±3.54 abcd	10.13±2.17	24.29±3.45 bc	6.13±1.73	86.21% (25/29)
5	0.27±0.08	20.58±7.60 bc	10.75±1.60	23.64±3.93 abc	6.25±1.14	92.42% (61/66)
6	0.24±0.10	23.21±3.17 abc	9.86±2.03	24.64±4.14 abc	5.71±1.27	100% (81/81)
7	0.23±0.13	18.50±4.12 ab	10.60±2.22	23.50±4.12 abc	6.50±1.43	100% (59/59)
8	0.31±0.23	22.86±4.69 abcd	9.79±1.72	23.33±4.44 bcd	5.57±1.09	90.20% (92/102)
9	0.25±0.05	17.50±5.24 bcd	10.00±2.10	26.00±2.24 bc	5.33±1.21	86.36% (38/44)
10	0.25±0.12	18.24±4.98 ab	9.24±3.46	23.71±4.37 bcd	5.00±1.58	100% (114/114)
11	0.24±0.13	21.79±3.17 bcd	8.86±1.96	25.50±5.71 abc	5.29±1.44	100% (101/101)
12	0.28±0.19	15.27±4.56 abcd	11.55±3.80	24.00±3.16 abc	6.55±2.16	96.43% (81/84)
13	0.17±0.06	22.73±7.86 abc	10.55±2.30	25.50±5.17 abc	5.82±1.47	89.06% (57/64)
14	0.26±0.14	18.53±3.50 a	9.67±2.32	22.47±2.75 ab	5.67±1.40	100% (109/109)
15	0.30±0.19	17.17±3.19 bcd	9.33±2.10	21.42±3.09 ab	5.50±1.45	100% (104/104)
16	0.32±0.19	20.09±9.96 bc	9.45±2.50	22.40±3.34 ab	5.00±1.55	97.44% (76/78)
17	0.34±0.25	20.50±3.85 bc	9.08±1.51	24.58±2.87 bc	5.08±1.16	100% (103/103)
Mean value	0.25±0.15	20.76±4.99	9.93±2.31	24.22±4.31	5.69±1.42	96.61% (1225/1268)

Notes: Data are presented as mean ± SEM. Values in parentheses under "Qualified straw rate" indicate the number of qualified straws per total straws produced on that day. SMF: sperm motility before freezing; MFS: motility of frozen-thawed sperm. Different superscript letters within the same column indicate significant differences among vapor heights ($p < 0.05$)

Table S4. Family-level semen quality and storage inventory for the Yan goose cryobank (30 families).

Family	Raw ejaculate volume (mL)	SMF (%)	Pre-freeze concentration (×10⁸ /mL)	MFS (%)	Post-thaw concentration (×10⁸ /mL)	Straws banked (n)	Cryovials (n)
1	0.17±0.08	25.00±5.59	11.11±1.83	25.33±2.69	6.11±1.36	26	2
2	0.29±0.13	17.00±8.72	11.14±4.30	19.57±2.15	5.14±1.57	27	2
3	0.17±0.09	18.60±2.19	9.40±2.07	22.00±2.74	5.80±1.10	20	1
4	0.29±0.14	17.00±4.47	8.83±1.83	20.00±0.00	4.67±1.75	35	2
5	0.25±0.07	19.13±4.19	11.50±2.39	24.13±2.75	6.38±1.60	41	2
6	0.28±0.24	24.00±4.18	10.00±2.35	23.60±3.51	5.60±1.52	21	1
7	0.17±0.09	23.89±3.33	9.78±1.86	23.56±3.36	5.89±1.54	36	2
8	0.16±0.07	23.33±3.54	9.44±1.01	26.11±4.43	6.11±0.78	24	1
9	0.27±0.10	22.86±3.93	10.71±2.87	30.00±2.89	6.29±0.76	31	2
10	0.19±0.08	21.00±5.35	9.86±1.57	24.86±3.93	5.86±0.69	31	2

11	0.18±0.08	20.63±3.20	9.63±1.19	20.25±0.71	5.00±1.41	31	2
12	0.20±0.06	21.25±3.54	10.00±2.07	26.88±5.94	5.88±1.36	35	2
13	0.26±0.13	22.57±4.24	9.43±1.40	25.00±2.89	5.57±1.13	38	2
14	0.17±0.06	20.83±2.40	10.67±3.14	22.50±2.74	5.83±2.14	24	1
15	0.21±0.10	23.00±6.71	9.60±6.70	26.40±6.11	5.00±1.22	25	2
16	0.20±0.11	16.17±4.49	8.33±1.75	22.50±2.74	4.50±1.05	24	1
17	0.12±0.05	19.71±0.76	9.57±1.72	21.43±2.44	5.86±1.07	18	1
18	0.39±0.16	20.00±3.54	8.00±2.55	23.00±2.74	4.80±0.45	22	1
19	0.27±0.22	20.00±0.00	9.67±1.15	23.33±2.89	6.33±0.58	21	1
20	0.23±0.10	18.00±5.70	8.60±1.82	23.40±2.30	5.40±1.34	23	1
21	0.42±0.16	24.17±3.76	10.83±0.98	30.50±9.77	6.33±1.03	22	1
22	0.88±0.17	17.50±10.61	8.00±0.00	25.00±7.07	3.50±0.71	24	1
23	0.27±0.09	22.00±4.00	8.67±1.97	25.83±3.76	5.00±1.26	25	1
24	0.23±0.12	21.25±2.50	11.75±0.96	25.00±4.08	6.50±0.58	23	1
25	0.36±0.07	25.00±5.00	9.33±2.31	23.33±2.89	5.67±1.53	24	1
26	0.42±0.28	15.00±0.00	10.67±1.53	23.33±2.89	7.00±1.00	29	2
27	0.48±0.17	16.25±4.79	11.25±0.96	22.50±2.89	6.00±0.82	48	2
28	0.63±0.08	20.00±14.14	10.50±2.12	27.50±3.54	6.50±0.71	32	2
29	0.24±0.07	23.33±4.08	10.17±1.60	25.33±4.08	5.67±1.51	31	2
30	0.27±0.08	14.50±3.32	14.00±5.35	28.25±2.36	8.00±2.71	27	2
Total	—	—	—	—	—	838	46
Mean value	0.25±0.15	20.76±4.99	9.93±2.31	24.22±4.31	5.69±1.42	—	—

Notes: Data are presented as mean ± SEM. All semen was cryopreserved using the optimized DMF-based protocol. Dashes (—) indicate not applicable.
